# Delayed extensor pollicis longus tendon rupture after minimally displaced distal radius fracture: a report of two cases

**DOI:** 10.1097/RC9.0000000000000295

**Published:** 2026-02-26

**Authors:** Danh Huy Luu, Manh Khanh Nguyen, Duc Phong Chu, Giang Linh Nguyen, Van Hau Phan

**Affiliations:** a Department of Orthopaedics, Viet Duc University Hospital, Hanoi, Vietnam; b University of Medicine and Pharmacy - Vietnam National University, Hanoi, Vietnam; c Department of Anatomy, Hanoi Medical University, Ha Noi, Vietnam

**Keywords:** case reports, extensor pollicis longus, radius fractures, tendon grafting, tendon injuries

## Abstract

**Introduction:**

Extensor pollicis longus (EPL) tendon rupture is an uncommon but serious complication following nondisplaced or minimally displaced distal radius fractures.

**Case presentation:**

We present two cases of delayed EPL tendon rupture. A 61-year-old woman and a 41-year-old man both developed inability to extend the thumb several weeks after conservative treatment of minimally displaced distal radius fractures. Intraoperative findings revealed poor tendon quality and significant retraction. Both patients were managed with palmaris longus tendon graft using the Pulvertaft weave technique.

**Outcomes:**

At 10 months, PRWHE improved from 48 to 10, exceeding the ≈ 14-point MCID; Kapandji improved from 6 to 9 on the operated side and matched the contralateral hand.

**Conclusion:**

Rupture of the EPL tendon after minimally displaced distal radius fracture is a rare but clinically significant complication with major impact on hand function. Delayed EPL rupture may present 3–12 weeks after minimally displaced distal radius fractures; prompt recognition of progressive loss of active IP extension is essential. When direct repair is not feasible and stumps are available, palmaris longus intercalated grafting offers reliable recovery while preserving independent index extension.

## Introduction

Rupture of the extensor pollicis longus (EPL) tendon is a rare injury, usually occurring after non-displaced or minimally displaced distal radius fractures, gout, rheumatoid arthritis, etc^[^1–6^]^. The reported rate of EPL tendon rupture after conservative treatment of minimally displaced distal radius fractures ranges from 0.07% to 0.88%^[^2^]^. Tendon rupture usually occurs 3–12 weeks after fracture^[^2–5,7–9^]^.

We report two cases of EPL rupture after minimally displaced distal radius fractures to illustrate clinical presentation, surgical management, and outcomes, and to discuss underlying pathophysiological mechanisms.

This work has been reported in line with the SCARE 2025 criteria^[^10^]^.

**Take-home message**: Even minimally/nondisplaced distal radius fractures can lead to delayed EPL rupture within the first 3–12 weeks. Vigilant follow-up for progressive loss of active IP extension with preserved passive motion enables early diagnosis and timely reconstruction^[^2,5,6,9^]^.

HIGHLIGHTS

- Delayed extensor pollicis longus (EPL) rupture after minimally displaced distal radius fracture.
- Two cases treated with palmaris longus graft using Pulvertaft weave.
- Full thumb ROM and ~ 90% grip/pinch at 10-month follow-up.
- Callus near Lister’s tubercle may signal risk for EPL ischemia.
- Close monitoring within the first 3 months is critical.

## Case presentation

### Case 1

**Figure 2.**
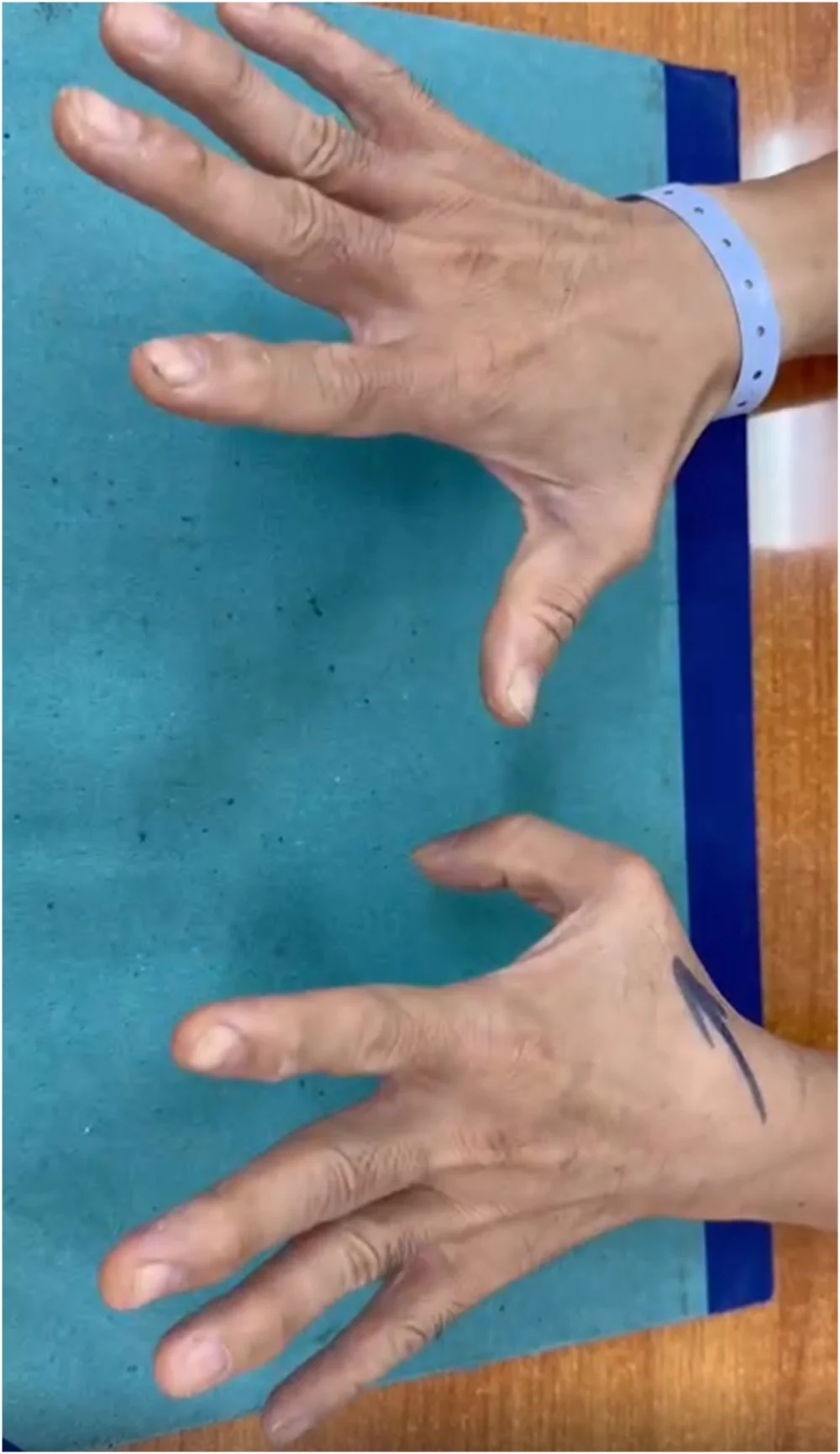
Preoperative radiographs showing minimally displaced distal radius fractures: (A) patient A (61/F); (B) patient B (41/M).

A 61-year-old woman, previously healthy and a homemaker, sustained a fall onto an outstretched hand one month prior. She did not seek medical care and self-managed with analgesics and anti-edema measures while continuing normal activities. She presented with a 4-day history of limited motion of the thumb. On examination, there was complete loss of active extension at the left thumb interphalangeal joint (positive “dropped thumb” sign) with preserved passive extension. Plain radiographs demonstrated a minimally displaced fracture of the distal radius on the left side with an atypical fracture line and a small, slightly displaced callus at the level of Lister’s tubercle (Fig. 2A).

### Case 2

**Figure 1.**
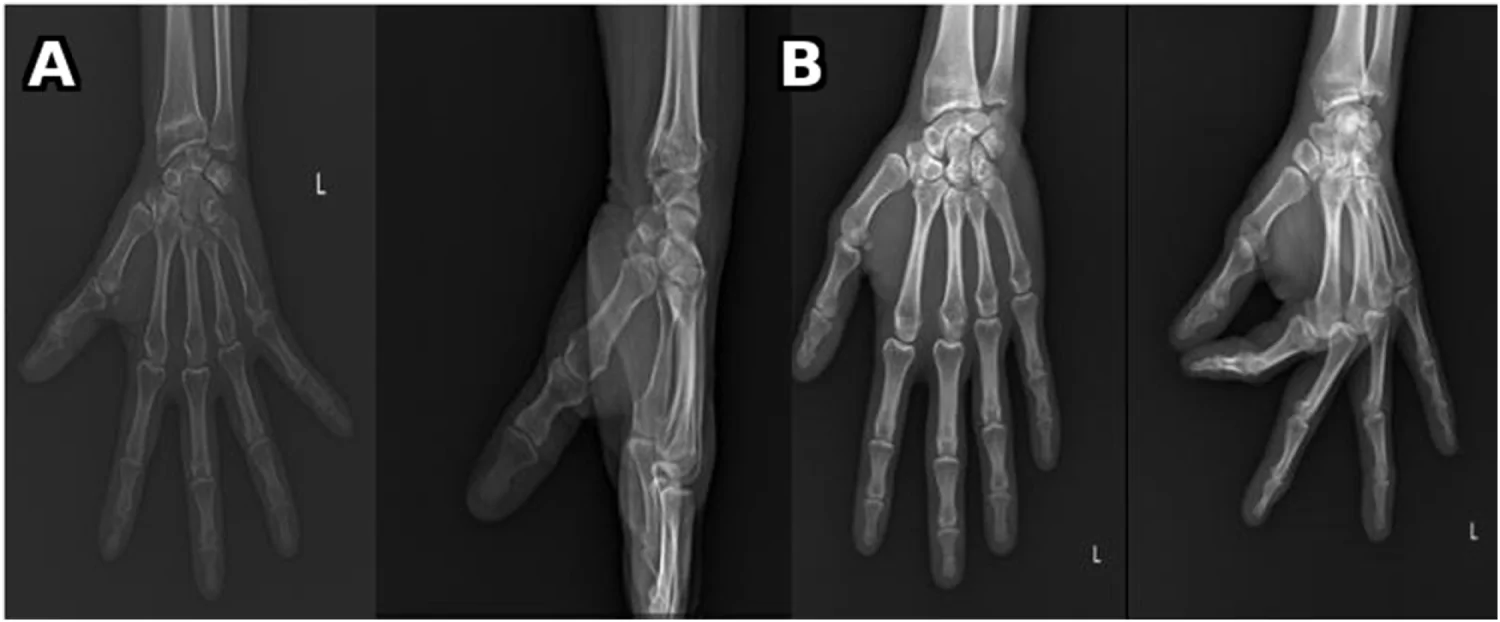
Preoperative photograph of patient B (41-year-old).

A 41-year-old man, previously healthy and working in business, fell from a motorcycle onto an outstretched hand 2 months earlier. He was treated with a short-arm cast for 4 weeks at 115 Nghe An Hospital and then underwent 5 weeks of rehabilitation without improvement of thumb extension. On examination, the thumb rested in increased flexion (Supplemental Digital Content Video S2, available at: http://links.lww.com/IJSCR/A15); he was unable to actively extend the interphalangeal joint (Fig. 1). There were no signs of inflammation (no swelling, warmth, erythema, or tenderness), and passive range of motion of all joints was full. Plain radiographs showed callus formation consistent with a minimally displaced distal radius fracture on the left side (Fig. 2B).

## Surgical technique

**Figure 3.**
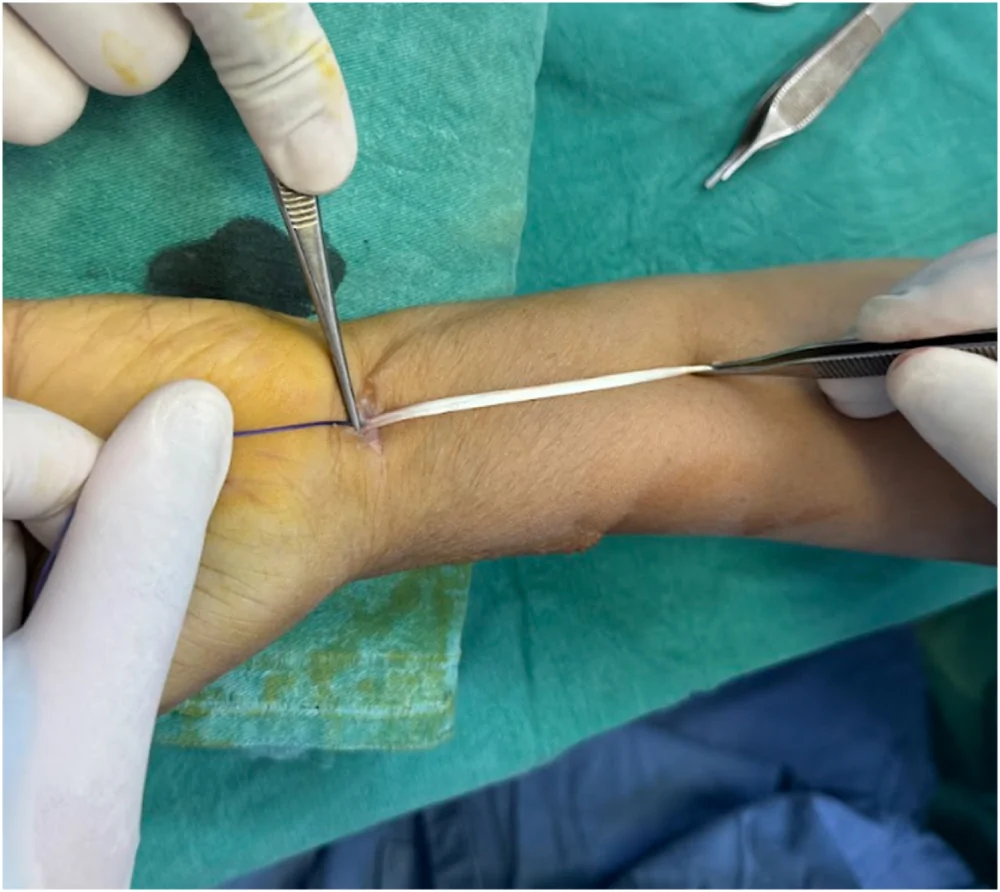
Intraoperative harvesting of palmaris longus tendon graft.

**Figure 4.**
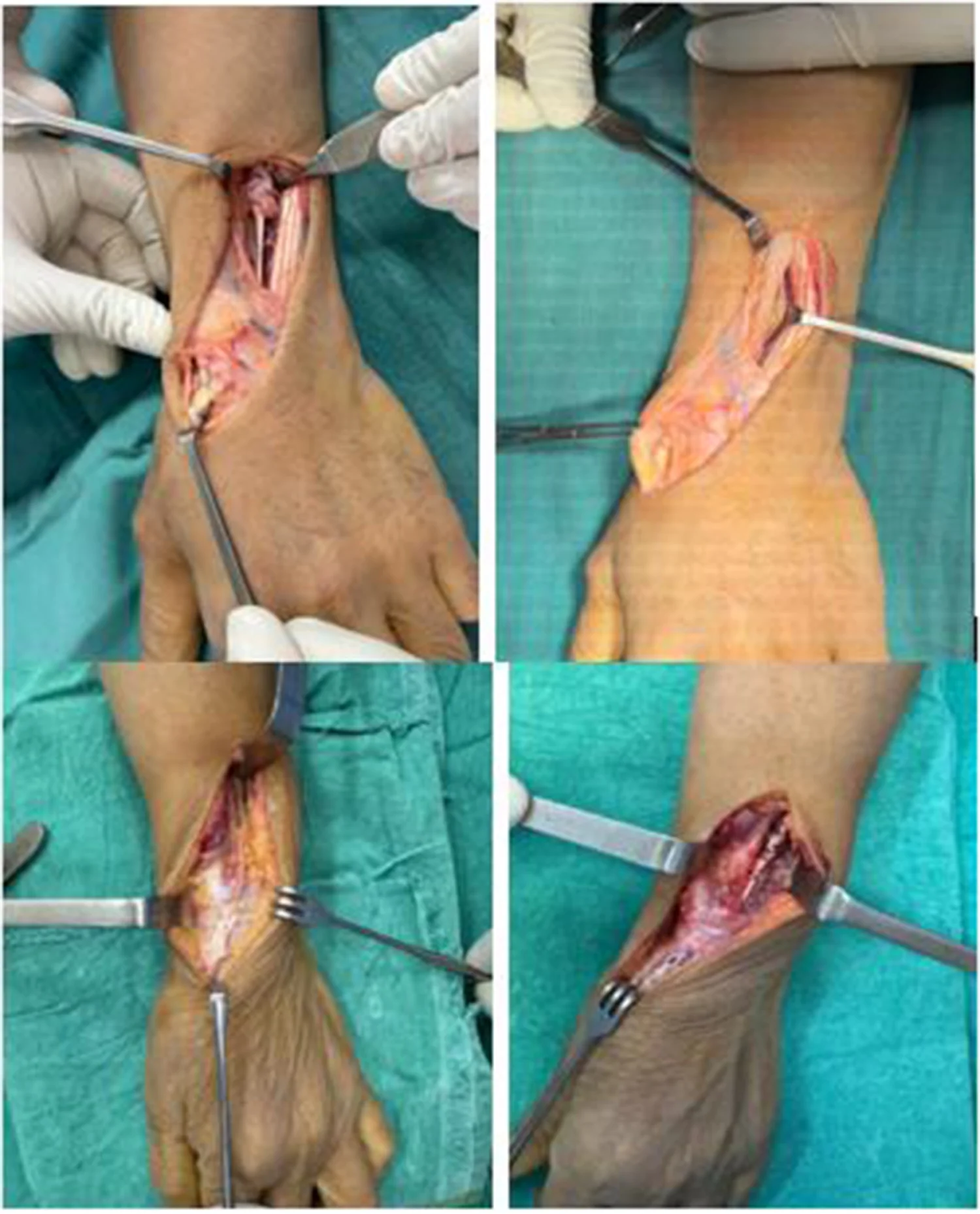
Intraoperative views: (A) ruptured EPL tendon with frayed ends; (B) after palmaris longus grafting using the Pulvertaft weave. EPL, extensor pollicis longus.

Both patients underwent surgical exploration through a 10–15-cm longitudinal incision along the EPL course at the wrist. The ruptured tendon ends were frayed and retracted (Fig. 4A), preventing direct repair. A palmaris longus tendon graft (8–10 cm) was harvested (Fig. 3) and interposed using the Pulvertaft weave technique (Fig. 4B). Graft tension was adjusted with the thumb in maximal extension. Postoperatively, the thumb was immobilized for 6 weeks before rehabilitation began.

Case 1: A small callus prominence adjacent to Lister’s tubercle was palpated and smoothed.

Case 2: No discrete bony spur was identified.

## Outcomes

Both patients were followed up at 2, 6, and 12 weeks, and every 3 months thereafter. Outcomes were assessed using the PRWHE (0–100, lower scores indicate better pain/ function) and the Kapandji opposition score (0–10, higher scores indicate better thumb opposition).

**Figure 5.**
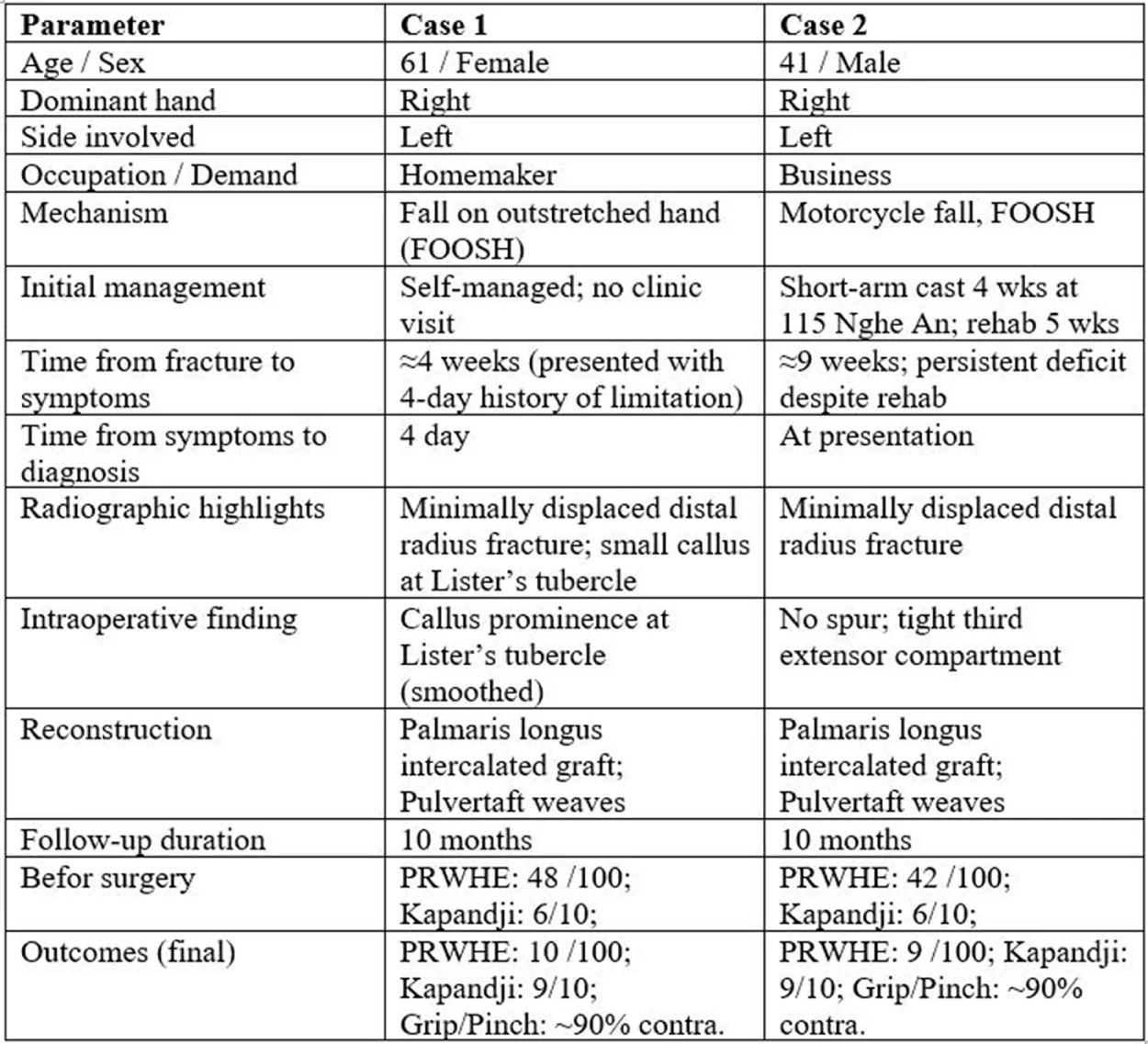
Summary table comparing the two cases.

At 10 months, PRWHE improved from 48 to 10, exceeding the ≈ 14-point MCID; Kapandji improved from 6 to 9 on the operated side and matched the contralateral hand (Fig. 5).

Active thumb interphalangeal extension at final follow-up is shown in Supplementary Video S1 (http://links.lww.com/IJSCR/A14).

## Discussion

Through the analysis of two clinical cases of delayed EPL tendon rupture following minimally displaced distal radius fractures, and in comparison with previous reports in the literature^[^1–3^]^, we identified both striking similarities and some notable differences. According to Skoff *et al*^[^5^]^, the incidence of EPL rupture in this group may reach 5%, which is significantly higher than in displaced fractures (0.3%–0.9%)^[^4,9^]^. This observation raises important questions regarding specific pathogenic mechanisms in minimally displaced fractures.

Reported incidence varies across series owing to heterogeneous cohorts and ascertainment methods, but most cases present several weeks after injury, commonly within weeks 3–12 from the index fracture^[^2,6^]^. Our two patients fit this window, presenting at approximately 4 and 9 weeks, respectively.

Ischemia of the EPL within a relatively tight third extensor compartment has been proposed as a key mechanism, especially when no sharp bony spur is present^[^2,5^]^. Local edema, hemarthrosis, or compartmental pressure may compromise the tendon’s microcirculation, predisposing to necrosis and rupture^[^5^]^. This mechanism aligns with our second case, in which no discrete bony prominence was found intraoperatively.

Mechanical abrasion over Lister’s tubercle can accelerate EPL wear, particularly when subtle malalignment or callus formation alters the tendon’s gliding path^[^2,5,7^]^. Prominent callus or hardware near the third compartment may further increase frictional load^[^2,5^]^. In our first case, a small callus prominence at Lister’s tubercle was identified and smoothed at surgery, supporting an attritional contribution.

Systemic factors – such as rheumatoid arthritis, corticosteroid exposure, or metabolic disease – can weaken tendon structure and impair healing. Neither patient in our series had known systemic risk factors, suggesting a predominantly local pathophysiology.

Early symptoms may be subtle (thumb weakness, vague dorsal wrist pain) and easily attributed to the fracture itself. A progressive loss of active IP extension with preserved passive motion is the key clinical sign and should prompt targeted evaluation for EPL rupture^[^9^]^. Plain radiographs may only show fracture healing/callus; ultrasound is useful to confirm discontinuity and assess tendon stump retraction when MRI is impractical^[^6^]^.

In conservatively managed minimally displaced distal radius fractures, clinicians should counsel patients that delayed EPL rupture can occur despite uneventful early recovery. We recommend scheduled reassessment between weeks 3–12, with instructions to return earlier for new or progressive loss of thumb IP extension.

This two-patient series limits generalizability, and we did not include a tendon-transfer comparison cohort. We used PRWHE and the Kapandji opposition score to capture patient-reported hand function and thumb opposition more directly. Larger prospective cohorts are needed to compare graft interposition and extensor indicis proprius (EIP) transfer in patient subgroups with different functional demands.

## Surgical methods

Surgical management of EPL tendon rupture can generally be classified into three main approaches: direct repair, tendon transfer, and tendon grafting. The choice of technique depends on the time of presentation, the extent of tendon damage, and the patient’s functional demands^[^1,5,8^]^.

Direct repair may be considered in acute cases when the rupture is identified early, the tendon ends are of good quality, and there is minimal retraction near the insertion. However, because EPL rupture typically occurs in a relatively avascular zone and often presents late, tendon ends are usually frayed and unsuitable for primary repair. As a result, this technique is rarely feasible in delayed cases.

Tendon transfer, most commonly using the EIP, remains the most frequently performed method due to its technical simplicity, shorter operative time, and reliable functional recovery^[^1,6^]^.

Autologous tendon grafting is another well-established method, particularly when there is significant tendon loss. The palmaris longus is the graft of choice in most cases. Although EIP transfer has been shown t/o yield excellent results, tendon grafting has the advantage of sparing adjacent tendons and preserving index finger function^[^11^]^.

In our two patients, both presented late with severely retracted tendon ends; therefore, we performed palmaris longus tendon grafting in each case. Functional outcomes at 6 months and at the most recent follow-up at 10 months were excellent. Importantly, none of the long-term complications sometimes associated with tendon grafting – such as limited thumb extension or index finger weakness^[^1^]^ – were observed. Both patients reported that their thumb function was “almost normal,” underscoring the adaptability of the graft in restoring hand function.

We selected intercalated palmaris longus grafting to restore native EPL excursion and preserve independent index finger extension. EIP-to-EPL transfer is reliable but may reduce independent index extension in high-demand users. In both cases, the proximal and distal EPL stumps were identifiable and the third compartment pulley intact – factors favoring graft interposition. Cosmesis was acceptable with a short wrist incision and a small PL harvest incision.

## Conclusion

Delayed rupture of the EPL can follow even minimally displaced distal radius fractures and leads to meaningful loss of hand function. Vigilant follow-up in the first 3 months and prompt evaluation of progressive loss of active IP extension with preserved passive motion enable early diagnosis. When primary repair is not feasible and tendon stumps are available, intercalated palmaris longus grafting provides reliable recovery; EIP-to-EPL transfer is a sound alternative when stumps are not retrievable. Clear monitoring pathways and tailored reconstruction help restore function and optimize outcomes.

## Data Availability

Not applicable.
